# Atypical STING-Vasculopathy Phenotype: Definite Usual Interstitial Pneumonia (UIP)-Pattern CT With Mixed Fibrotic Histology: A Case Report

**DOI:** 10.7759/cureus.90302

**Published:** 2025-08-17

**Authors:** Arya Kermanshah, Aman Aher, Jochen Gerstner Saucedo, Urvi Kawade, Yasamin Mirzabeigi, Pritish Aher

**Affiliations:** 1 Internal Medicine, St. John's Riverside Hospital, Yonkers, USA; 2 Nutrition and Exercise Physiology, University of Missouri, Columbia, USA; 3 Diagnostic Radiology, University of Colorado Anschutz Medical Campus, Aurora, USA; 4 Medicine, Pravara Institute of Medical Sciences, Loni, IND; 5 Pathology, University of Miami Miller School of Medicine, Jackson Memorial Hospital, Miami, USA; 6 Radiology, University of Miami Miller School of Medicine, Miami, USA

**Keywords:** interstitial lung disease, livedo reticularis, lung transplantation, mixed pattern fibrosis, nonspecific interstitial pneumonia, sting-associated vasculopathy with onset in infancy, usual interstitial pattern, vasculopathy, viral respiratory tract infection

## Abstract

STING‑associated vasculopathy with onset in infancy (SAVI) is a rare autoinflammatory disorder that causes systemic inflammation, vasculopathy, and progressive interstitial lung disease (ILD). The pulmonary manifestations of SAVI typically resemble a nonspecific interstitial pneumonia (NSIP) pattern both radiologically and histologically. We present a case of a 20‑year‑old male with genetically confirmed SAVI who developed acute hypoxic respiratory failure triggered by respiratory syncytial virus infection, despite appropriate treatment. Imaging revealed extensive subpleural fibrosis, traction bronchiectasis, and honeycombing. These findings were observed in the setting of a CT appearance strongly consistent with a definite usual interstitial pneumonia (UIP) pattern. Ultimately, the patient underwent a lung transplant, and histopathology of the explanted lungs revealed fibrotic changes, including diffuse interstitial thickening, lymphoid aggregates, and honeycombing, without definitive fibroblastic foci, indicating a mixed UIP and NSIP pattern of fibrosis. This case illustrates a rare UIP‑pattern imaging phenotype in SAVI‑associated ILD, despite a mixed UIP‑NSIP histologic pattern of pulmonary fibrosis, and highlights the potential for acute viral infection to precipitate decompensation in patients with interferon‑driven pulmonary fibrosis.

Written informed consent was obtained from the patient for publication of this report and accompanying images.

## Introduction

STING‑associated vasculopathy with onset in infancy (SAVI) is an autoinflammatory disorder caused by gain‑of‑function mutations in TMEM173, resulting in constitutive STING activation and chronic type I interferon signaling. The estimated incidence is unknown due to the rarity of the condition, but under 30 cases have been reported in the literature, with both autosomal dominant and, more rarely, autosomal recessive inheritance patterns described [1,2]. The disease is characterized by early-onset systemic inflammation, cutaneous vasculopathy, including livedo reticularis, telangiectasias, ulcerations, and acral necrosis. It also causes failure to thrive and progressive interstitial lung disease, which may lead to respiratory failure and lung transplantation [2-4]. Additional systemic features include recurrent low‑grade fevers, lymphadenopathy, polyarthritis, myositis, and, less commonly, neurologic or renal involvement.

SAVI‑associated interstitial lung disease (ILD) represents the major cause of morbidity and mortality in these patients. Patients typically present with chronic cough, tachypnea, hypoxemia, and progressive dyspnea, with a nonspecific interstitial pneumonia (NSIP)‑like pattern on imaging, characterized by ground‑glass opacities, reticulation, and bronchiectasis, while lacking the basal‑predominant honeycombing characteristic of classic usual interstitial pneumonia (UIP) [2]. Laboratory findings typically include elevated acute‑phase reactants, increased interferon‑stimulated gene expression, and autoantibody positivity (notably rheumatoid factor) [2]. Histological analysis is not required for diagnosis but may show diffuse lymphocytic infiltration, bronchus-associated lymphoid tissue hyperplasia, and mild fibrosis predominate, with classical UIP features, such as fibroblastic foci, honeycombing, and patchy scarring, rarely observed [2,5,6].

Management of SAVI is challenging, as conventional immunosuppressive therapies (e.g., corticosteroids, methotrexate, tumor necrosis factor inhibitors) are generally ineffective [2,7,8]. Janus kinase (JAK) inhibitors, especially ruxolitinib and tofacitinib, have demonstrated significant potential, with early treatment linked to better outcomes, particularly in pulmonary disease [2,9,10]. However, responses may vary, and progression to advanced lung disease may occur, necessitating lung transplantation [11,12]. The long‑term prognosis for patients with pulmonary fibrosis is uncertain, as life expectancy largely depends on the severity and progression of the disease, along with any related complications the patient might experience [4,13].

This case is notable for presenting with radiological UIP‑pattern pulmonary fibrosis and histological mixed UIP‑NSIP pattern ILD in a patient with SAVI [1,2]. Additionally, the patient’s RSV‑triggered acute decompensation and favorable response to multimodal immunosuppression followed by lung transplantation contrasts with the usual progressive, treatment‑refractory course observed in SAVI‑related ILD [1,9]. Overall, this case broadens the radiologic and histologic spectrum of SAVI‑associated lung disease and challenges the assumption that this condition is uniformly NSIP‑like by demonstrating radiologic UIP features, underscoring broader phenotypic variability. Given the clinical overlap and variability in histologic findings, high‑resolution CT remains the cornerstone for non‑invasive ILD characterization in interferonopathies such as SAVI.

## Case presentation

A 20‑year‑old male with a confirmed TMEM173‑mutation SAVI presented in December 2024 with worsening shortness of breath. He reported a nine‑day history of flu‑like symptoms (fatigue, sinus congestion, sore throat, and cough) followed by low‑grade fevers and progressive dyspnea. He had been treated as an outpatient with levofloxacin without improvement and now required 8-10 L/min oxygen via nasal cannula to maintain oxygen saturation ≥90 % (baseline 6-8 L/min).

Past medical history included asthma, environmental allergies, and gastro‑esophageal reflux secondary to an unrepaired hiatal hernia, as well as intermittent arthritis and skin ulcerations attributed to SAVI. Medications were baricitinib, monthly anifrolumab, and a prednisone taper (alternating 20 mg/5 mg every other day).

On arrival, he was tachypneic (>20 breaths/min), cyanotic with minimal exertion, and had diffuse bilateral inspiratory crackles. Arterial blood gas on high-flow nasal cannula (HFNC) 30 L/40% fraction of inspired oxygen showed pH: 7.39, partial pressure of arterial oxygen: 52 mm Hg, and partial pressure of arterial carbon dioxide: 46 mm Hg. He was admitted to the ICU for hypoxemic respiratory failure.

Despite HFNC escalation to 60 L/60%, prednisone taper to 10 mg/5 mg, and baricitinib increase to 4 mg daily, oxygen requirements worsened. Polymerase chain reaction (PCR) was positive for respiratory syncytial virus (RSV); ribavirin 600 mg PO q8h was initiated. Weekly PCRs soon became negative, yet HFNC support (20 L/60%) remained necessary.

**Figure 1:**
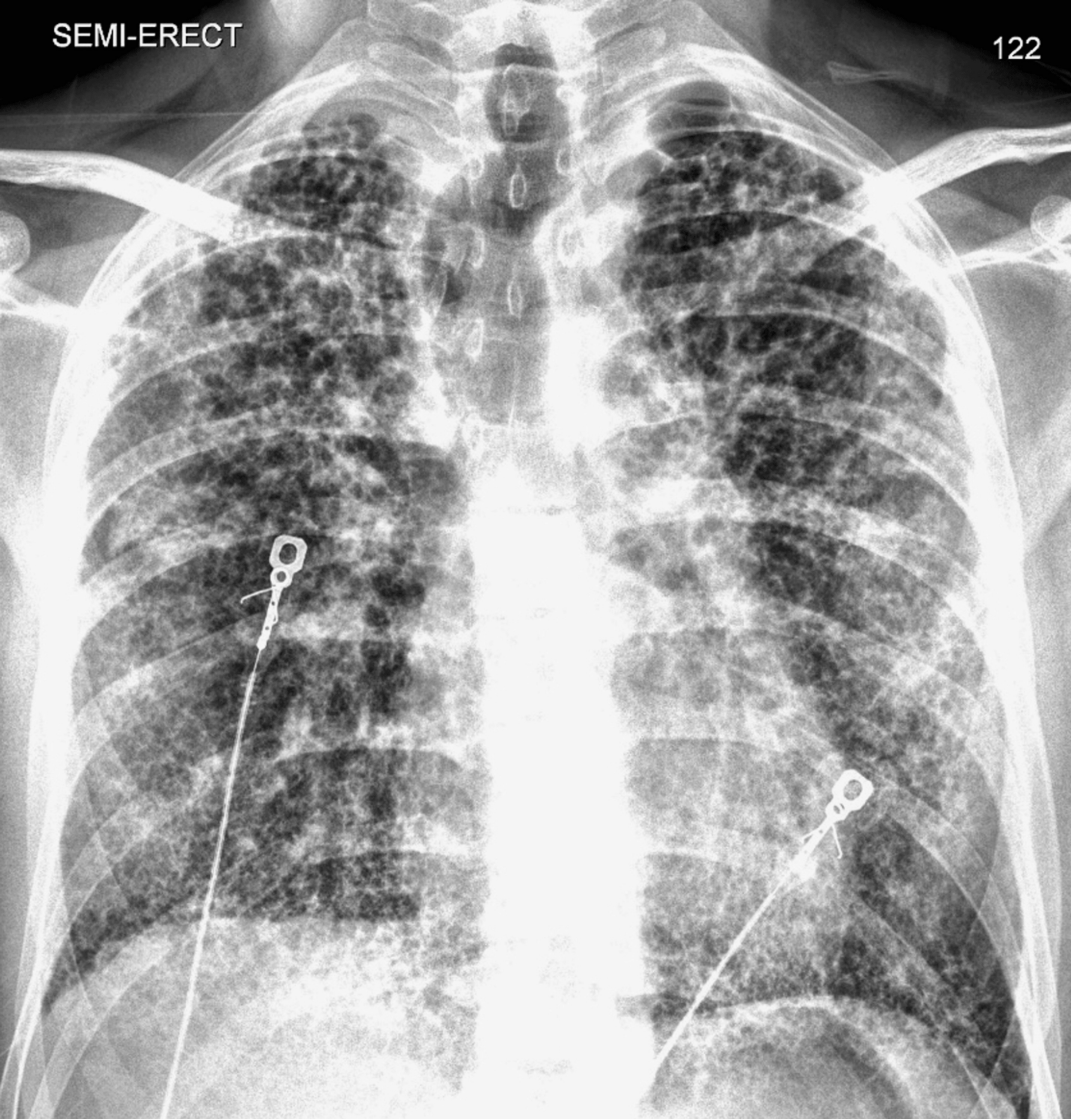
Anterior-posterior chest radiograph Frontal chest radiographs demonstrate bilateral coarse interstitial opacities with a heterogeneous, reticulonodular pattern. Findings are consistent with advanced fibrotic lung disease in the setting of SAVI-associated ILD. ILD: Interstitial lung disease; SAVI: STING‑associated vasculopathy with onset in infancy

**Figure 2:**
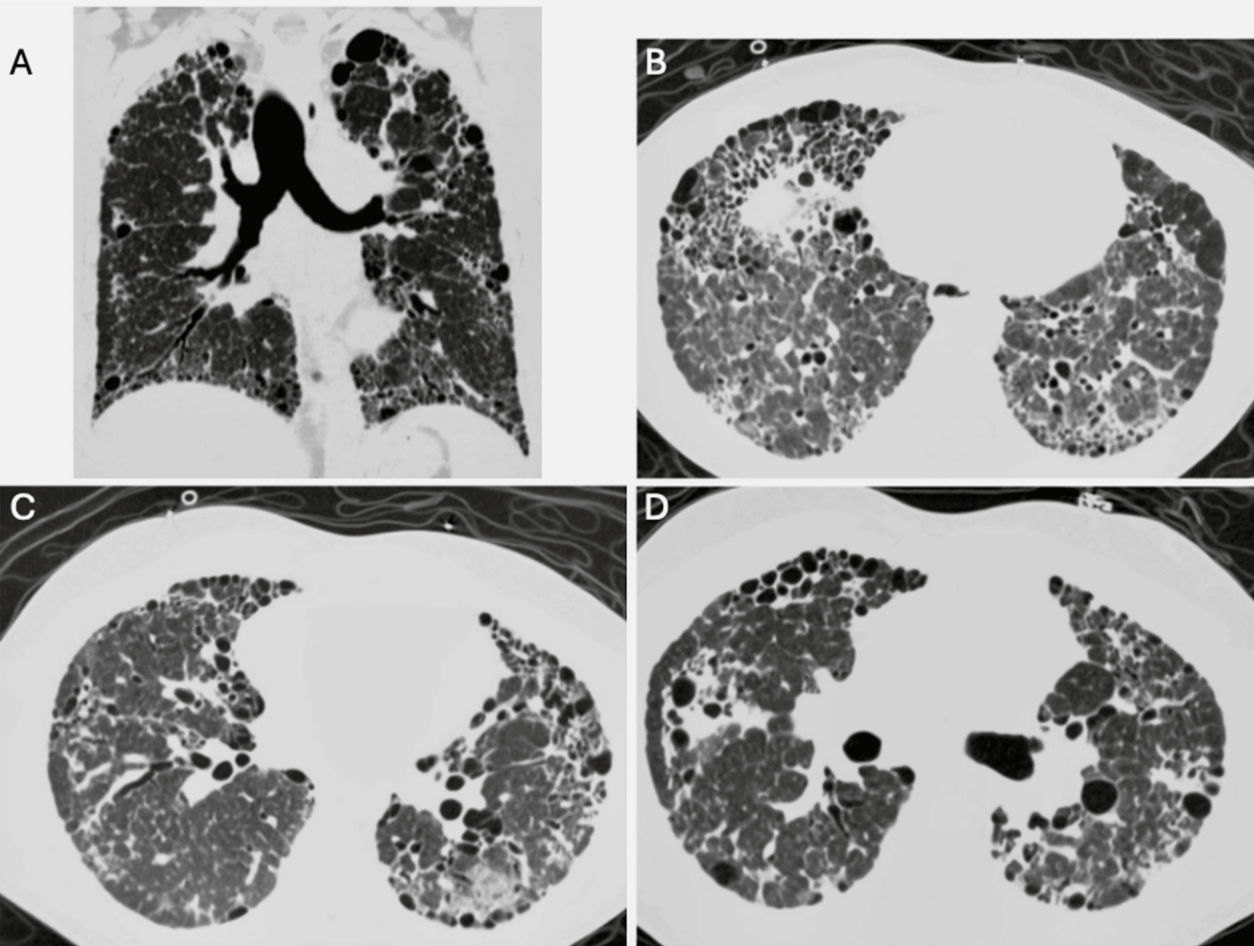
High-resolution CT chest (A) Coronal view: Bilateral diffuse reticulation with traction bronchiectasis, cystic changes, and honeycombing, most prominent in the lower lobes. Relative subpleural sparing is noted. (B-D) Axial views: Advanced fibrotic changes with bilateral basal-predominant honeycombing, traction bronchiectasis, and pulmonary cysts. Scattered ground-glass opacities are present with areas of relative subpleural sparing, consistent with UIP pattern per Fleischner criteria. UIP: Usual interstitial pneumonia

Chest radiograph (Figure 1) showed diffuse interstitial opacities overlying fibrotic lung. High‑resolution CT revealed bilateral lower‑lobe‑predominant reticulation with traction bronchiectasis, architectural distortion, and honeycombing with multiple cysts of varying sizes bilaterally (Figure 2); findings consistent with a definite UIP pattern, atypical for SAVI. Differential considerations included RSV pneumonia, superimposed bacterial infection, pulmonary embolism, and acute ILD exacerbation; RSV‑triggered decompensation was favored.

The patient was listed for bilateral lung transplantation. He underwent transplant with venoarterial extracorporeal membrane oxygenation support three months after admission; the postoperative course included osteomyelitis and mild primary graft dysfunction but no rejection. Immunosuppression comprised basiliximab, corticosteroids, mycophenolate, and continuation of anifrolumab. He was discharged on a slow oxygen‑wean protocol.

**Figure 3:**
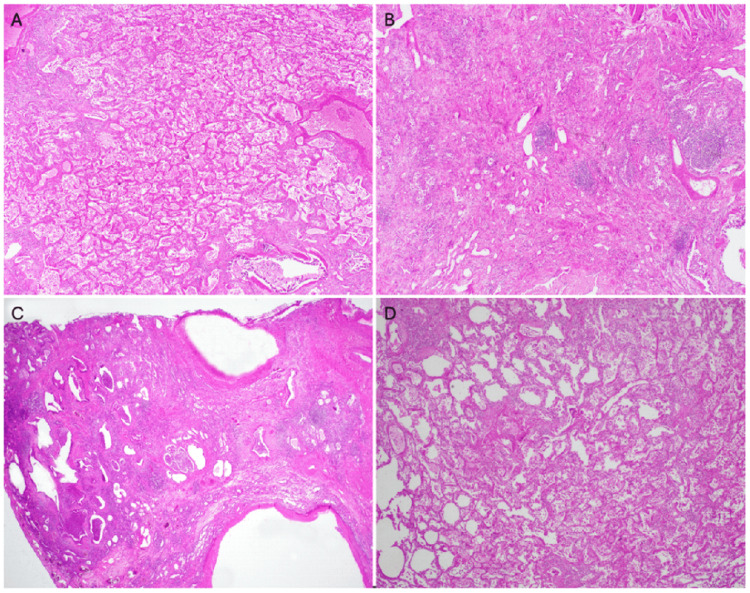
Explanted lung histology (H&E, 5×) (A) Diffuse fibrotic remodeling without discrete fibroblastic foci or true honeycomb cysts. (B) Predominantly uniform interstitial thickening with capillary proliferation and lymphoid aggregates. (C) Honeycombing features next to a large blood vessel, a feature more typical of UIP. (D) Patchy interstitial fibrosis with areas of preserved alveolar architecture, more typical of NSIP. H&E: Hematoxylin and eosin; NSIP: Nonspecific interstitial pneumonia; UIP: Usual interstitial pneumonia

Histopathology of explanted lungs (Figure 3) demonstrated heterogeneous fibrosis with features of both UIP and NSIP patterns: honeycomb cysts, diffuse interstitial thickening, and lymphoid aggregates, correlating with the imaging‑pathology mismatch.

Written informed consent was obtained from the patient for publication of this case and associated images.

## Discussion

SAVI is marked by excessive inflammation affecting skin, vasculature, and lungs. Inappropriate activation of the cyclic GMP-AMP synthase-stimulator of interferon genes (cGAS-STING) pathway drives chronic type I interferon signaling, leading to endothelial injury, lymphocytic infiltration, and fibrosis [2,3]. The pathogenesis of SAVI is driven by unregulated cytosolic DNA sensing through the cGAS‑STING pathway, resulting in immune‑mediated tissue injury, including endothelial dysfunction [2,3,14]

Diagnostic work-up combines clinical features, chronic inflammatory markers, interferon-stimulated gene expression, and genetic confirmation [2,15]. Chemokine (C-X-C motif) ligand 9 and chemokine (C-X-C motif) ligand 10 levels are often elevated but may be less pronounced in UIP‑pattern cases, limiting biomarker utility in advanced or atypical disease [16].

Imaging in SAVI‑ILD classically mirrors NSIP, whereas UIP‑pattern honeycombing is rare [2,17,18]. In our patient, CT fulfilled Fleischner criteria for definite UIP, yet explant histology showed mixed UIP‑NSIP fibrosis, underscoring that chronic immune‑mediated injury can produce composite patterns [19,20]. This discordance supports a pragmatic reliance on high-resolution computed tomography (HRCT) for transplant referral when pathology is unobtainable or non‑diagnostic.

Traditional immunosuppression yields limited pulmonary benefit [2]. JAK inhibitors mitigate systemic inflammation but may not halt fibrotic progression [11]. Our patient’s need for transplant despite baricitinib reflects this limitation. Given potential CT-histology mismatch, surgical lung biopsy or transbronchial cryobiopsy may be useful in select cases where subtype confirmation (UIP vs. NSIP) would materially change management [19,20].

RSV likely precipitated the acute decline, antiviral efficacy remains uncertain, and immunomodulation increases viral susceptibility. Awareness of viral triggers is, therefore, key to early escalation of care.

## Conclusions

This case of SAVI with atypical UIP‑pattern ILD expands the recognized radiologic and histopathologic spectrum of monogenic interferonopathies. While NSIP features are typically described in SAVI, it demonstrates that HRCT may provide more reliable ILD characterization than histology alone, particularly for transplant timing and prognosis. By documenting the first CT-defined definite UIP pattern in a genetically confirmed SAVI patient, our report underscores the critical importance of systematic high-resolution imaging in interferonopathies. The striking CT-pathology discordance underlines the pitfalls of relying solely on explant histology and supports a proactive, imaging‑driven strategy for monitoring disease trajectory and transplant candidacy. Future multicenter registries and longitudinal imaging cohorts are warranted to detail the full phenotypic spectrum, refine the timing of advanced interventions (JAK inhibitors, antifibrotics, and transplantation), and evaluate post‑transplant recurrence‑mitigation strategies. Early recognition of such atypical presentations will enable more personalized management, optimize surgical outcomes, and inform trials aimed at interrupting interferon‑driven fibrotic remodeling in SAVI and related disorders.
